# The safety and efficacy of tislelizumab, alone or in combination with chemotherapy, for the treatment of non-small cell lung cancer: a systematic review of clinical trials

**DOI:** 10.1186/s12890-023-02755-3

**Published:** 2023-12-08

**Authors:** Amin Daei Sorkhabi, Mahta ZareDini, Asra Fazlollahi, Aila Sarkesh, Amirreza Naseri, Seyed Ehsan Mousavi, Seyed Aria Nejadghaderi, Mark J M Sullman, Ali-Asghar Kolahi, Saeid Safiri

**Affiliations:** 1 Student Research Committee, Tabriz University of Medical Sciences, Tabriz, Iran; 2 Hematology and Oncology Research Center, Tabriz University of Medical Sciences, Tabriz, Iran; 3 Neurosciences Research Center, Aging Research Institute, Tabriz University of Medical Sciences, Tabriz, Iran; 4 Research Center for Evidence-based Medicine, Iranian EBM Centre: A Joanna Briggs Institute (JBI) Center of Excellence, Tabriz University of Medical Sciences, Tabriz, Iran; 5 Tuberculosis and Lung Diseases Research Centre, Tabriz University of Medical Sciences, Tabriz, Iran; 6 School of Medicine, Shahid Beheshti University of Medical Sciences, Tehran, Iran; 7 Systematic Review and Meta-analysis Expert Group (SRMEG), Universal Scientific Education and Research Network (USERN), Tehran, Iran; 8 Department of Life and Health Sciences, University of Nicosia, Nicosia, Cyprus; 9 Department of Social Sciences, University of Nicosia, Nicosia, Cyprus; 10 Social Determinants of Health Research Center, Shahid Beheshti University of Medical Sciences, Tehran, Iran; 11 Clinical Research Development Unit of Tabriz Valiasr Hospital, Tabriz University of Medical Sciences, Tabriz, Iran

**Keywords:** Tislelizumab, Anti-PD-1 monoclonal antibody, Immune checkpoint inhibitors, NSCLC, Lung Cancer, Systematic review

## Abstract

**Background:**

Tislelizumab is an anti-programmed death-1 (PD-1) monoclonal antibody with a construction that enables it to have a higher affinity to its target. We aimed to evaluate tislelizumab’s safety and efficacy for treating non-small cell lung cancer (NSCLC).

**Methods:**

Embase, Scopus, PubMed, Web of Science, and Google Scholar were searched up to December 20, 2022. The review only included randomized controlled trials (RCTs) that evaluated the safety or efficacy of tislelizumab for treating patients with lung cancer. The revised Cochrane risk-of-bias tool (RoB2) was utilized to evaluate study quality.

**Results:**

There were four RCTs identified, which included 1565 patients with confirmed locally advanced or metastatic squamous and/or non-squamous types of NSCLC. Treatment with tislelizumab was associated with better progression-free survival (PFS) and objective response rate (ORR), particularly when used in combination with chemotherapy. Almost all patients in both arms reported at least one treatment-emergent adverse event (TEAE). Decreased hematologic indexes accounted for more than 20% of the grade ≥ 3 TEAEs in the tislelizumab plus chemotherapy group. The proportion of TEAE that led to death in the tislelizumab plus chemotherapy arms ranged from 3.2 to 4.2%. Hypothyroidism, pneumonitis, and hyperglycemia were the most frequently noted immune-mediated adverse events in the tislelizumab group.

**Conclusions:**

Tislelizumab, whether used alone or in combination with chemotherapy, seems to demonstrate both a safety and efficacy as a treatment for NSCLC.

**Supplementary Information:**

The online version contains supplementary material available at 10.1186/s12890-023-02755-3.

## Introduction

Globally, lung cancer ranks as the second most prevalent form of cancer and the primary cause of cancer-related death [1]. Moreover, lung cancer presents one of the poorest prognoses, with five-year survival rates ranging from 4 to 17%, owing to the asymptomatic progression and the absence of adequate screening measures [2]. Pathologically, lung cancer is divided into small cell lung cancer (SCLC) and non–small cell lung cancer (NSCLC), representing 15% and 85% of cases, respectively [3]. In addition, NSCLC can be further categorized into adenocarcinoma, squamous cell carcinoma, and large cell carcinoma [3]. The appropriate treatment varies according to the pathological characteristics, but normally involves surgical resection and chemoradiation. However, despite substantial medical advancements, reducing mortality from lung cancer remains challenging [4, 5]. Conventional chemotherapeutic agents exhibit a lack of specificity and limited pharmacokinetic properties, due to their lipophilic nature and rapid first-pass metabolism, resulting in non-targeted effects on healthy tissues that evoke undesirable consequences and hinder therapeutic efficacy [4, 5]. Thus, research has shifted from chemical-induced cytotoxic therapeutics to genetic modification-guided targeted therapies and programmed death-1 (PD-1) / programmed death ligand 1 (PD-L1)-based immunotherapies.

As well as counteracting cancer-related immunosuppression and enhancing antigen presentation, chemotherapy medications enhance the PD-L1 expression implicated in chemoresistance, through the synergistic combination of the PD-1/PD-L1 axis blockade with standard chemotherapeutic regimens [6, 7]. Immunotherapy has been adopted in routine clinical practice for NSCLC, since the initial report about the objective response to PD-1 inhibition in 2012 and the approval of nivolumab by the Food and Drug Administration in 2015 [8, 9]. Although the clinical efficacy and safety profiles of the anti-PD-1/PD-L1 therapeutics currently used to treat lung cancer are promising, a large proportion of patients are unresponsive or eventually progress, which highlights the need for further research into novel agents targeting the PD-1/PD-L1 axis [10].

Tislelizumab (BGB-A317) is a humanized immunoglobulin G4-variant anti-PD-1 monoclonal antibody [11]. It has been shown to have high efficacy and an acceptable safety profile for several tumor types and is currently authorized in China for treating advanced squamous and non-squamous NSCLCs, as well as hepatocellular carcinoma (HCC), esophageal squamous cell carcinoma (ESCC), urothelial carcinoma, and classical Hodgkin’s lymphoma (cHL) [11, 12]. In comparison to other anti-PD-1 agents (i.e., nivolumab and pembrolizumab), tislelizumab exhibits a stronger affinity for PD-1 and an off-rate that is 50 times slower than nivolumab and 100 times slower than pembrolizumab [13]. The higher binding affinity can be partially explained by the fact that tislelizumab binds to PD-1 in a different orientation than other anti-PD-1 agents, with a binding region on PD-1 that partially overlaps pembrolizumab’s but differs substantially from nivolumab’s [13]. Moreover, unlike other anti-PD-1 agents, tislelizumab has a unique construction with a neutralized Fc domain of the antibody that enables it to inhibit binding to the FcγR on macrophages and antibody-mediated phagocytosis, while also enhancing T cell activity, all of which partially overcomes the resistance associated with anti-PD-1 therapies [14, 15].

Given the potential advantages of tislelizumab over currently employed anti-PD-1 agents, we performed a systematic review of randomized controlled trials to assess tislelizumab’s safety and efficacy in treating lung cancer, both alone and in combination with chemotherapy.

## Methods

This systematic review followed the guidelines outlined in the Preferred Reporting Items for Systematic Reviews and Meta-Analysis (PRISMA) 2020 [16].

### Literature search

We searched Embase, Scopus, PubMed, and the Web of Science, without time or language restrictions, up to December 20, 2022. In addition, we manually searched the initial 300 results from the Google Scholar search engine and conducted backward/forward citation searches within the included studies to identify any additional relevant papers. The search was performed using phrases linked to tislelizumab or BGB-A317 in all areas of the scientific literature and lung neoplasms in the title and abstract. The comprehensive search strategy is depicted in Table S1.

### Study selection

The studies found through the systematic search were exported to EndNote 20 and all duplicate records were eliminated. Following this, two researchers individually reviewed title/abstract of each publication, according to the inclusion/exclusion criteria. Both researchers independently assessed the full texts of all screened papers, and any discrepancies were resolved through discussion or consultation with a third researcher. The inclusion criteria were that they must be randomized control trials (RCTs) evaluating the safety or efficacy of tislelizumab, either as monotherapy or combined with standard supportive care, for the treatment of lung cancer of any stage, in comparison to a placebo or the best supportive care. The exclusion criteria were the following: (1) studies that were not RCTs, animal studies, in vitro studies, perspectives, opinions, case reports, case series, notes, news, books, book chapters, meeting abstracts, editorials, letters, commentaries, review articles, meta-analyses, retracted articles, and re-analyses of previously published articles; (2) studies enrolling healthy individuals or those with disorders that were not lung cancer; and (3) studies that were investigating therapeutic approaches other than tislelizumab.

### Data extraction

Two researchers autonomously extracted the data utilizing a standard data extraction sheet in Microsoft Office Excel, and two additional authors independently verified all of the extracted data. The extracted information included: (1) study demographics, including the name of the first author, study title, publication year, RCT phase, and sample size; (2) participant characteristics, including the age range, sex ratio, smoking status, Eastern Cooperative Oncology Group (ECOG) performance status of the patients, expression of PD-L1, cancer ascertainment and characteristics; (3) medication characteristics; and (4) the main outcomes of the studies, including the safety and efficacy of the medication.

### Quality assessment

The same two researchers independently assessed the included studies’ utilizing version 2 of the Cochrane risk-of-bias tool (RoB 2) [17] for RCTs. Discrepancies were resolved by discussion or consultation with a third researcher. In summary, RoB 2 assesses the quality of studies across five types of bias, including bias resulting from the randomization process, deviations from the intended intervention, missing outcome data, outcome measurement, and the selection of the reported results [17]. Bias domains were recorded as having a “low,“ “high,“ or “some concern” [17].

## Results

### Study selection

**Fig. 1:**
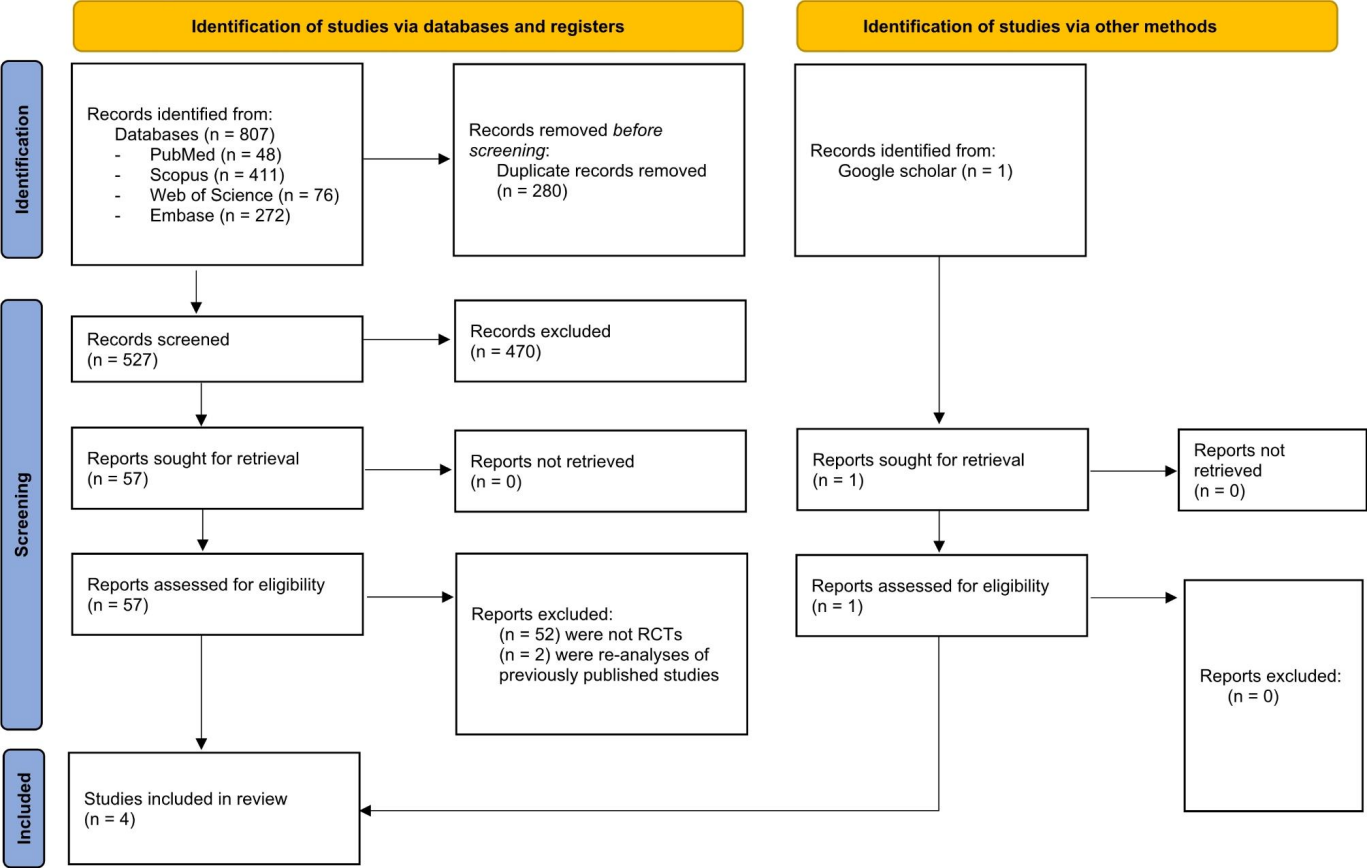
Study selection process

The systematic search of the literature found 808 records, but 280 of those were excluded as duplicate records, and using the title and abstract a further 470 studies were excluded. The full texts of the remaining 58 publications underwent screening, but 54 of those were excluded (52 were not RCTs and two were re-analyses of previously published studies). Finally, four RCTs met the inclusion criteria [18–21] (Fig. 1), but the low number of studies and the considerable heterogeneity in them, particularly in terms of the subtypes of NSCLC, interventions, and subjects in the control groups, prevented a meta-analysis from being undertaken.

### Quality assessment

**Fig. 2:**
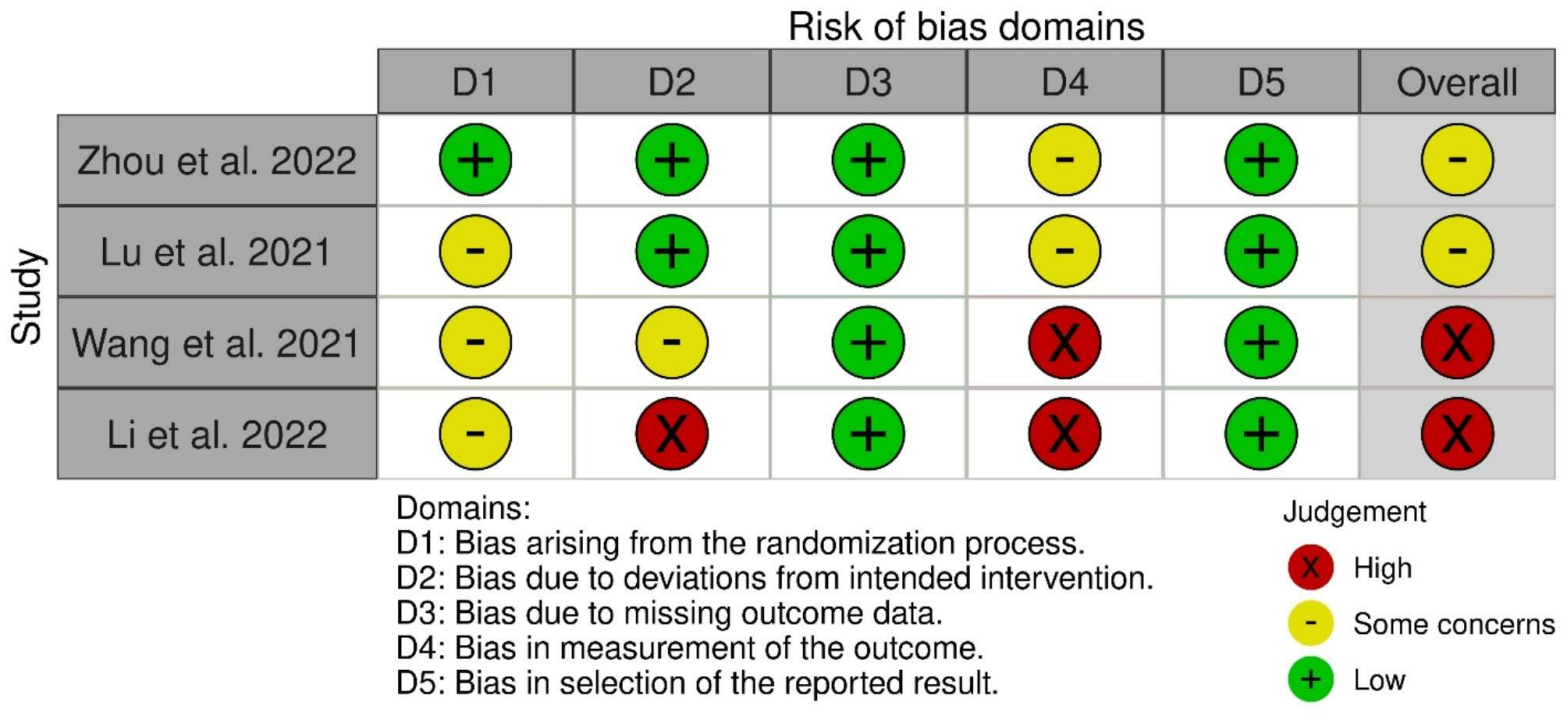
Summary of the risk of bias assessments for the included studies

There was a consistent low risk of bias in missing outcome data and the selection of reported results across all four RCTs. However, the quality level varied in the deviations from the intended intervention criteria. Two studies showed some concerns regarding outcome measurement bias, while the other two demonstrated high risk level. Altogether, half of the trials had a high risk of bias [18, 20] and the other half had some concern [19, 21] (Fig. 2 and Table S2).

### Study characteristics

The included studies were published over the period 2021–2022. Studies were carried out on people with confirmed locally advanced (stage IIIB) or metastatic (stage IV) squamous and/or non-squamous types of NSCLC, with only one study focusing on advanced epidermal growth factor receptor (EGFR) + TP53 co-variant lung adenocarcinomas. As a first-line treatment, tislelizumab plus chemotherapy was compared with chemotherapy alone. In addition, as a second/third-line treatment tislelizumab was compared with docetaxel in one study [21]. Tislelizumab was delivered intravenously (200 mg) every three weeks for 4 to 10 cycles. The chemotherapy regimen was comprised of pemetrexed, paclitaxel, or docetaxel with/without platinum-based drugs (carboplatin or cisplatin). The participants were aged from 25 to 88 years old (Tables 1 and 2).

**Table 1:** Baseline characteristics of the included studies

Study ID	Country	Study design	Phase	Groups	N	Tislelizumab dosage	Concomitant chemotherapy	Follow-up (median)
Li et al. 2022 [18]	China	RCT	N/A	TS + CT CT	33 33	200 mg IV OD for 3 weeks in a 3-week cycle for 6 cycles	Mixed 500 mg/m2 pemetrexed disodium with 100 ml of normal saline and mixed 25 mg/m2 of cisplatin with 250 ml of normal saline for 6 cycles	N/A
Lu et al. 2021 [19]	China	RCT	III	TS + CT CT	223 111	200 mg IV once every 3 weeks for 4 to 6 cycles	Platinum-based chemotherapy (carboplatin AUC 5 or cisplatin 75 mg/m2 in combination with pemetrexed 500 mg/m2), every 3 weeks for 4 to 6 cycles	9.8 months (95% CI: 9.23–10.38)
Wang et al. 0.2021 [20]	China	RCT	III	Arm A: TS + CT (PTX + CBP) Arm B: TS + CT (nab PTX + CBP) Arm C: CT (PTX + CBP)	120 119 121	200 mg IV (day 1) every 3 week	Paclitaxel (175 mg/m2 IV, day 1) or nab paclitaxel (100 mg/m2, days 1, 8, and 15); and carboplatin (AUC of 5, day 1), every 3 week	8.6 months (95% CI: 8.1- 9.0 months)
Zhou et al. 2022 [21]	Russia, Poland, Mexico, Brazil, New Zealand, China, Turkey, Bulgaria, Lithuania, Slovakia	RCT	III	TS CT	535 270	200 mg IV every 3 weeks	Docetaxel 75 mg/m2 IV, every 3 weeks	TS: 16.0 months (range: 0.3–43.5 months) CT: 10.7 months (range: 0.03–38.3 months)

Abbreviations: RCT: randomized controlled trial; TS: tislelizumab; CT: chemotherapy; PTX: paclitaxel; CBP: carboplatin; IV: intravenous; OD: once daily; AUC: area under the curve; CI: confidence interval

**Table 2:** Characteristics of the participants in the included studies

Study ID	Groups	Number	Male (%)	Age (median)	Smoking Status			Histopathology				ECOG performance status		Tumor stage	
					Former	Current	Never	SCC	ADC	ASC	Other	0	1	Locally advanced (IIIB)	Metastatic (IV)
Li et al. 2022 [18]	TS + CT CT	33 33	15 (45.4) 14 (42.4)	65.74 (59–78) 65.58 (58–78)	N/A N/A	N/A N/A	N/A N/A	0 0	33 (100) 33 (100)	0 0	0 0	N/A N/A	N/A N/A	N/A N/A	N/A N/A
Lu et al. 2021 [19]	TS + CT CT	223 111	168 (75.3) 79 (71.2)	60 (27–75) 61 (25–74)	115 (51.6) 53 (47.7)	32 (14.3) 13 (11.7)	76 (34.1) 45 (40.5)	N/A N/A	215 (96.4) 107 (96.4)	1 (0.4) 2 (1.8)	7 (3.1) 2 (1.8)	54 (24.2) 24 (21.6)	169 (75.8) 87 (78.4)	40 (17.9) 21 (18.9)	183 (82.1) 90 (81.1)
Wang et al. 2021 [20]	Arm A: TS + CT (PTX + CBP) Arm B: TS + CT (nab PTX + CBP) Arm C: CT (PTX + CBP)	120 119 121	107 (89.2) 112 (94.1) 111 (91.7)	60 (41–74) 63 (38–74) 62 (34–74)	96 (80.0) 107 (89.9) 98 (81.0)		24 (20.0) 12 (10.1) 23 (19.0)	TS + CT: 239 (100) CT: 121 (100)	N/A N/A	N/A N/A	N/A N/A	31 (25.8) 22 (18.5) 32 (26.4)	89 (74.2) 97 (81.5) 89 (73.6)	38 (31.7) 40 (33.6) 44 (36.4)	82 (68.3) 79 (66.4) 77 (63.6)
Zhou et al. 2022 [21]	TS CT	535 270	416 (77.8) 206 (76.3)	61.0 (28–88) 61.0 (32–81)	373 (69.7) 188 (69.6)		162 (30.3) 82 (30.4)	248 (46.4) 122 (45.2)	N/A N/A	N/A N/A	N/A N/A	116 (21.7) 50 (18.5)	419 (78.3) 220 (81.5)	84 (15.7) 33 (12.2)	451 (84.3) 237 (87.8)

Abbreviations: TS: tislelizumab; CT: chemotherapy; PTX: paclitaxel; CBP: carboplatin; SCC: squamous cell carcinoma; ADC: adenocarcinoma; ASC: adenosquamous carcinoma; ECOG: Eastern Cooperative Oncology Group; N/A: not available

### Efficacy

The efficacy results are shown in Table 3. Taken together, all investigations supported the efficacy of tislelizumab through much higher progression-free survival (PFS) and a higher objective response rate (ORR). In addition, the overall survival (OS) [21], duration of response (DoR) [19–21], and disease control rate [18, 21] were all considerably better when tislelizumab was included.

**Table 3:** Efficacy outcome measurements for the included studies

Study ID	Groups	N	ORR (%) (95% CI)	Median PFS, months (95% CI)	DoR, months (95% CI)	DCR, n (%)
Li et al. 2022 [18]	TS + CT CT	33 33	60.61% 33.33%	12.12 (8.50-13.91) 7.65 (3.88–10.52)	N/A N/A	27 (81.82) 19 (57.58)
Lu et al. 2021 [19]	TS + CT CT	223 111	57.4 (50.6–64.0) 36.9 (28.0–46.6)	9.7 (7.7–11.5) 7.6 (5.6–8.0) Hazard ratio = 0.65 (0.46–0.90), *p* = 0.004	8.5 (6.80–10.58) 6.0 (4.99–not estimable)	N/A N/A
Wang et al. 2021 [20]	Arm A: TS + CT (PTX + CBP) Arm B: TS + CT (nab PTX + CBP) Arm C: CT (PTX + CBP)	120 119 121	73 (63.6–80.3) 75 (66.0-82.3) 50 (40.4–58.8)	7.6 (6.0-9.8) 7.6 (5.8–11.0) 5.5 (4.2–5.7) A versus C: Hazard ratio = 0.52 (0.37–0.74), *p* < 0.001 B versus C: Hazard ratio = 0.48 (0.34–0.68), *p* < 0.001	8.2 (5.0-not estimable) 8.6 (6.3-not estimable) 4.2 (2.8–4.9)	N/A N/A
Zhou et al. 2022 [21]	TS CT	535 270	22.6 (19.1–26.4) 7.1 (4.3–10.8)	4.2 (3.9–5.5) 2.6 (2.2–3.8) Hazard ratio = 0.63 (0.53–0.75), *p* < 0.0001	13.5 (8.5–19.6) 6.0 (2.1–7.2)	298 (55.70) 114 42.20

Abbreviations: TS: tislelizumab; CT: chemotherapy; PTX: paclitaxel; CBP: carboplatin; ORR: objective response rate; PFS: progression-free survival; DoR: duration of response; DCR: disease control rate; CI: confidence interval; N/A: not available

#### Progression-free survival

PFS is defined as the period of time, both during and after treatment, in which there is no progression or worsening of the disease. All studies reported significant improvements in the PFS for patients treated with tislelizumab, whether as a standalone treatment or combined with chemotherapy, when compared to the chemotherapy-only group. One study reported a median PFS of up to 12.12 months in patients with advanced EGFR + TP53 co-variant lung adenocarcinoma, when treated with tislelizumab plus chemotherapy [18]. In subgroup analyses, PFS was significantly improved in both stage IIIB and stage IV diseases [19, 20]. Furthermore, Lu et al. demonstrated median PFS of 9.0 and 7.6 months (hazard ratio = 0.66 [95% CI: 0.32 to 1.38]) in stage IIIB disease, as well as 9.7 and 7.5 months (hazard ratio = 0.63 [95% CI: 0.44 to 0.92]) in stage IV disease for patients receiving tislelizumab plus chemotherapy and chemotherapy alone [19]. Likewise, Wang et al. reported median PFS of 9.8 and 5.6 months (hazard ratio = 0.40 [95% CI: 0.22 to 0.75]) in arms A versus C, and 11 and 5.6 months (hazard ratio = 0.37 [95% CI: 0.20 to 0.69]) in arms B versus C in stage IIIB disease, as well as 7.6 and 5.2 months (hazard ratio = 0.57 [95% CI: 0.38 to 0.86]) in arms A versus C, and 7.4 and 5.2 months (hazard ratio = 0.54 [95% CI: 0.35 to 0.82]) in arm B versus C in stage IV disease [20].

There was some inconsistency between the findings of Lu et al. and Wang et al., regarding the effects of tumor cell PD-1 expression of less than 1% on the efficacy of tislelizumab for treating of lung cancer [19, 20]. According to Lu et al., patients with tumor cell PD-L1 expression levels of 50% or more had higher PFS (hazard ratio = 0.34 [95% CI: 0.19 to 0.61]), while the study failed to draw any conclusions for patients with tumor cell PD-L1 expression levels that were below 1% (hazard ratio = 0.73 [95% CI: 0.46 to 1.18]) or between 1% and 49% (hazard ratio = 1.10 [95% CI: 0.53 to 2.28]). The p-value for the interaction between the three mentioned groups was 0.03 [19]. In contrast, Wang et al. found PFS improvements across all PD-L1 subgroups, with a trend towards a more significant PFS advantage in the PD-L1-positive subgroup at the 1% cutoff (hazard ratios for PD-L1 ≥ 1%: PD-L1 < 1% ratio were 0.72 [95% CI: 0.36 to 1.46], *p* = 0.37) and 0.53 [95% CI: 0.26 to 1.07], *p* = 0.07) for arms A versus C and arms B versus C, respectively). However, interaction analyses were unable to identify any predictive effects of PD-L1 for a PFS advantage from combination treatments [20]. Similarly, in the study by Zhou et al. PD-L1 expression of ≥ 25% was found to improve PFS (hazard ratio = 0.37 [95% CI: 0.28 to 0.49], *p* < 0.0001) in those with lung cancer who were treated with tislelizumab [21].

#### Objective response rate

Measurement of the ORR is one method for assessing the efficacy of a new treatment and this involves calculating the percentage of patients that have a partial (tumor shrinks) or complete (tumor disappears) response to the treatment. Significantly improved ORRs were reported in all studies and ranged from 22.6% in the tislelizumab monotherapy group (second/third-line treatment) to 75% in the tislelizumab plus nab-paclitaxel group. In the tislelizumab monotherapy arm, the PD-L1 of ≥ 25% led to an ORR of 37.4%, which contrasts with 22.6% at any level of PD-L1 expression [21].

### Safety

The safety findings are shown in Table 4 and Table S3. Almost all of the patients in both arms reported at least one treatment-emergent adverse event (TEAE) [19–21]. The most common TEAEs in the tislelizumab arm were decreased hematologic indexes (anemia, neutropenia, thrombocytopenia, and leukopenia), increased alanine transaminase (ALT), increased aspartate transaminase (AST), nausea, and decreased appetite, but there were slightly fewer TEAEs than in the control arm [19–21]. In terms of severity, most TEAEs were grades 1 to 2. In the study by Zhou et al., TEAEs of grade ≥ 3 severity were found in 42.1% of the tislelizumab group and 74.8% of the docetaxel group [21]. However, this pattern was not found in the other studies [19, 20]. Decreased neutrophil levels, decreased white blood cell count, neutropenia, and leukopenia (grade ≥ 3) were observed in more than 20% of the patients treated with tislelizumab plus chemotherapy, which was consistent with the chemotherapy-only arm [19, 20]. Furthermore, serious TEAEs were found in 33.3–38.1% of the tislelizumab plus chemotherapy arm, leading to the discontinuation of treatment in 12.5–29.7% and death in 3.2–4.2% of the patients [19, 20]. In the tislelizumab monotherapy group, 6.4% of patients died due to TEAEs [21]. Hypothyroidism (7.9–8.6%), pneumonitis (4.5–9.0%), and hyperglycemia emerged as the most common immune-mediated AEs in the tislelizumab group, which were mostly grade 1 to 2 in severity [19–21].

**Table 4:** Safety outcomes for the included studies

First Author	Groups	Patients with ≥ 1 TEAE, n (%)	Grade ≥ 3 TEAEs, n (%)	Serious TEAEs, n (%)	TEAEs leading to death, n (%)	TEAEs leading to discontinuation, n (%)	TEAEs leading to dose modification or treatment delays, n (%)
Li et al. 2022 [18]	TS + CT CT	N/A N/A	N/A N/A	N/A N/A	N/A N/A	N/A N/A	N/A N/A
Lu et al. 2021 [19]	TS + CT CT	222 (100) 109 (99.1)	150 (67.6) 59 (53.6)	74 (33.3) 23 (20.9)	7 (3.2) 2 (1.8)	57 (25.7) 10 (9.1)	149 (67.1) 57 (51.8)
Wang et al. 2021 [20]	Arm A: TS + CT (PTX + CBP) Arm B: TS + CT (nab PTX + CBP) Arm C: CT (PTX + CBP)	120 (100.0) 117 (99.2) 117 (100.0)	106 (88.3) 102 (86.4) 98 (83.8)	44 (36.7) 45 (38.1) 29 (24.8)	4 (3.3) 5 (4.2) 5 (4.3)	15 (12.5) 35 (29.7) 18 (15.4)	N/A N/A N/A
Zhou et al. 2022 [21]	TS CT	517 (96.8) 254 (98.4)	225 (42.1) 193 (74.8)	184 (34.5) 84 (32.6)	34 (6.4) 12 (4.7)	64 (12.0) 34 (13.2)	125 (23.4) 96 (37.2)

Abbreviations: TEAE: treatment-emergent adverse event; TS: tislelizumab; CT: chemotherapy; PTX: paclitaxel; CBP: carboplatin; N/A: not available

## Discussion

The primary aim of this systematic review was to examine the efficacy and safety of tislelizumab for treating NSCLC, based on the results of RCTs. The limited available evidence suggests that tislelizumab improves OS in patients with NSCLC, in comparison to docetaxel [22]. In addition, tislelizumab plus chemotherapy can significantly improve PFS and ORR, in comparison to chemotherapy alone, with a comparable safety profile [23, 24].

From a pathophysiological point of view, the tumor microenvironment and immune escape, which are crucial for its growth and development, are facilitated by PD-1 and PD-L1 [25]. PD-1 is expressed on the surface of lymphocytes, and research has found that the PD-1 gene is an appropriate marker for predicting outcomes in lung cancer [26]. Anti-PD-1 and anti-PD-L1 immunotherapeutic strategies have shown dramatically improved outcomes in patients diagnosed with NSCLC [27]. A systematic review of RCTs found that PD-L1 inhibitors were effective in some malignancies, including NSCLC and SCLC [4]. Furthermore, a comparison of PD-1 and PD-L1 inhibitors in a meta-analysis found that PD-1 inhibitors were more effective for treating advanced NSCLC [28].

As an anti-PD-1 monoclonal immunoglobulin G4 antibody, tislelizumab is an immunotherapeutic anti-neoplastic drug that has been approved in China for treating cHL [29] and has shown efficacy in treating multiple solid tumors, such as ESCC [22], gastric/gastroesophageal junction adenocarcinoma [30], and lung cancer. To the best of our knowledge, only four RCTs have examined the safety and efficacy of tislelizumab for treating patients with different subtypes of NSCLC, despite this being the most prevalent type of lung cancer [31]. No RCTs were found that investigated the efficacy of tislelizumab for treating patients with SCLCs.

The safety of tislelizumab in patients with lung cancer has been investigated in multiple phase 1 clinical trials [22, 29]. Furthermore, phase 2 clinical trials of tislelizumab, combined with chemotherapy, found that this treatment regimen was effective for treating advanced lung cancer [32], and NSCLC [33]. In a phase 3 clinical trial, Zhou et al. found that tislelizumab was effective in treating patients with locally advanced or metastatic squamous or non-squamous NSCLC, irrespective of PD-L1 expression [21]. This study was the only available RCT that compared the efficacy of tislelizumab monotherapy with chemotherapy alone. Moreover, in another phase 3 clinical trial, Wang et al. found that adding tislelizumab to chemotherapy improved outcomes and had manageable TEAEs in patients with squamous NSCLC [24]. In a re-analysis of this study, they reported that adding tislelizumab to platinum-based chemotherapy led to improvements in the patients’ health-related quality of life [24]. Furthermore, Lu et al. found that adding tislelizumab to chemotherapy could be a new first-line treatment option for advanced non-squamous NSCLC, irrespective of disease stage, with improved outcomes and comparable safety [19]. In addition, the researchers reported an improvement in the patients’ health-related quality of life [19]. Finally, Li et al. found that when combined with pemetrexed, tislelizumab was an effective and safe treatment for advanced EGFR + TP53 co-variant lung adenocarcinoma [18]. The abovementioned studies found that a combination of tislelizumab and chemotherapy had substantial efficacy and safety for treating different subtypes of NSCLC.

In addition to the safety and efficacy of tislelizumab, studies have also evaluated its cost-effectiveness in the Chinese healthcare system. Compared with conventional docetaxel chemotherapy, tislelizumab was found to be a cost-effective treatment strategy in advanced or metastatic NSCLC beyond the first-line setting [23], which was also found in previously treated advanced NSCLC patients [34]. Another study reported that adding tislelizumab to first-line chemotherapy was cost-effective, regardless of the baseline characteristics of those with locally advanced or metastatic non-squamous NSCLC [23], while Liang et al. found that tislelizumab plus chemotherapy was a cost-effective approach to the first-line treatment of advanced non-squamous NSCLC [35]. These findings indicated the need for future cost-effectiveness research on the use of tislelizumab in other settings.

This present study represents the first attempt to systematically review the safety and efficacy of tislelizumab therapy in patients with NSCLC using the highest quality evidence. In addition to its novelty, the main strengths of the present study included the comprehensive coverage obtained by searching five major sources and using the PRISMA-guided approach. The main limitations included the small number of RCTs and the substantial risk of bias in these studies. In addition, three of the four included RCTs were conducted in China, which restricts the generalizability of the findings to other populations. Similarly, the investigation of different subtypes of NSCLC within the included RCTs, along with the significant heterogeneity among them - particularly in terms of interventions and the characteristics of participants in the control groups - constituted additional limitations. These constraints precluded conducting a meta-analysis or additional subgroup analyses, thereby making the findings of these studies somewhat less conclusive. Despite the abovementioned limitations, the results of this systematic review support the use of tislelizumab, whether on its own or in combination with chemotherapy, as an effective and safe treatment for NSCLC. These findings underscore the necessity for additional well-designed RCTs in this field.

## Conclusions

This systematic review found that tislelizumab monotherapy, and its combination with standard chemotherapy, were both effective and safe for treating NSCLC. However, due to the shortcomings of this evidence, we suggest caution in the clinical application of these findings. Further studies on the efficacy and safety of tislelizumab in anti-tumor therapy are highly recommended.

### Electronic supplementary material

Below is the link to the electronic supplementary material.

Supplementary Material 1

## Data Availability

The data that support the findings of this study are available from the corresponding author, upon reasonable request.

## Abbreviations

DoR: duration of response
HQoI: health-related quality of life
NSCLC: non–small cell lung cancer
ORR: objective response rate
OS: overall survival
PD-1: programmed death 1
PD-L1: programmed death ligand 1
PFS: progression-free survival
PRISMA: Preferred Reporting Items for Systematic Reviews and Meta-Analysis
RCT: randomized control trial
RoB 2: Risk of Bias 2
SCLC: small cell lung cancer
TEAE: treatment-emergent adverse event
