# Supplementary material for: The safety and efficacy of tislelizumab, alone or in combination with chemotherapy, for the treatment of non-small cell lung cancer: a systematic review of clinical trials
Source: BMC Pulm Med. 2023 Dec 8;23:495. doi: 10.1186/s12890-023-02755-3 (PMC10704633; doi:10.1186/s12890-023-02755-3)
Supplement: Supplementary file 1 — Supplementary Material 1 [file 12890_2023_2755_MOESM1_ESM.docx]

**Safety and efficacy of tislelizumab for the treatment of lung cancer: A systematic review of clinical trials**

**Table S1.** Search strategy for each database and search engine

| **Database**  **(search date)** | **Step** | **Search strategy** | **Number of results** |
| --- | --- | --- | --- |
| PubMed  (12.20.2022) | #1 | “Tislelizumab”[Supplementary Concept] OR “Tislelizumab”[all] OR “BGB-A317”[all] | 137 |
|  | #2 | “Lung Neoplasms”[mh] OR “Pulmonary Blastoma”[mh] OR ((“Lung”[mh] OR “Lung*”[tiab] OR “Pulmonary”[tiab]) AND (“Neoplasms”[mh] OR “Neoplas*”[tiab] OR “Cancer*”[tiab] OR “Tumor*”[tiab] OR “Tumour*”[tiab] OR “Malignan*”[tiab] OR “Carcinoma*”[tiab] OR “Carcinoid*” OR “Adenocarcinoma*”[tiab] OR “Lymphoma*”[tiab] OR “Sarcoma*”[tiab] OR “blastoma*”[tiab] OR “Fibrosarcoma*”[tiab] OR “Leiomyosarcoma*”[tiab])) | 478,263 |
|  | #3 | #1 AND #2 | 48 |
| Scopus  (12.20.2022) | #1 | ALL ( “Tislelizumab” OR “BGB-A317” ) | 1,054 |
|  | #2 | TITLE-ABS-KEY ( (“Lung*” OR “Pulmonary”) AND (“Neoplas*” OR “Cancer*” OR “Tumor*” OR “Tumour*” OR “Malignan*” OR “Carcinoma*” OR “Carcinoid*” OR “Adenocarcinoma*” OR “Lymphoma*” OR “Sarcoma*” OR “blastoma*” OR “Fibrosarcoma*” OR “Leiomyosarcoma*”) ) | 701,819 |
|  | #3 | #1 AND #2 | 411 |
| Web of Science  (12.20.2022) | #1 | ALL=(“Tislelizumab” OR “BGB-A317”) | 308 |
|  | #2 | TS=((“Lung*” OR “Pulmonary”) AND (“Neoplas*” OR “Cancer*” OR “Tumor*” OR “Tumour*” OR “Malignan*” OR “Carcinoma*” OR “Carcinoid*” OR “Adenocarcinoma*” OR “Lymphoma*” OR “Sarcoma*” OR “blastoma*” OR “Fibrosarcoma*” OR “Leiomyosarcoma*”)) | 520,195 |
|  | #3 | #1 AND #2 | 76 |
| Embase  (12.20.2022) | #1 | 'tislelizumab'/exp OR 'tislelizumab' OR 'bgb-a317'/exp OR 'bgb-a317' | 890 |
|  | #2 | ('neoplas*':ti,ab,kw OR 'cancer*':ti,ab,kw OR 'tumor*':ti,ab,kw OR 'tumour*':ti,ab,kw OR 'malignan*':ti,ab,kw OR 'carcinoma*':ti,ab,kw OR 'carcinoid*':ti,ab,kw OR 'adenocarcinoma*':ti,ab,kw OR 'lymphoma*':ti,ab,kw OR 'sarcoma*':ti,ab,kw OR 'blastoma*':ti,ab,kw OR 'fibrosarcoma*':ti,ab,kw OR 'leiomyosarcoma*':ti,ab,kw) AND ('lung*':ti,ab,kw OR 'pulmonary':ti,ab,kw) | 613,546 |
|  | #3 | #1 AND #2 | 272 |
| Google Scholar  (12.27.2022) |  | (“Tislelizumab” OR “BGB-A317”) AND (“Lung Cancer” OR “Pulmonary Cancer”) | 2,185 |

**Table S2.** Quality assessment of the included studies.

| Study ID | D1 | D2 | D3 | D4 | D5 | Overall bias* |
| --- | --- | --- | --- | --- | --- | --- |
| Zhou et al. 2022 (1) | Low Risk | Low Risk | Low Risk | Some Concerns | Low Risk | Some Concerns |
| Lu et al. 2021 (2) | Some Concerns | Low Risk | Low Risk | Some Concerns | Low Risk | Some Concerns |
| Wang et al. 2021 (3) | Some Concerns | Some Concerns | Low Risk | High Risk | Low Risk | High Risk |
| Li et al. 2022 (4) | Some Concerns | High Risk | Low Risk | High Risk | Low Risk | High Risk |

**D1:** Bias arising from the randomization process

**D2:** Bias due to deviations from intended interventions

**D3:** Bias due to missing outcome data

**D4:** Bias in measurement of the outcome

**D5:** Bias in selection of the reported results

*RoB2 overall risk of bias judgment

Low risk of bias → The study is judged to be at low risk of bias for all domains for this result.

Some concerns → The study is judged to raise some concerns in at least one domain for this result, but not to be at high risk of bias for any domain.

High risk of bias → The study is judged to be at high risk of bias in at least one domain, or to have some concerns for multiple domains in a way that substantially lowers confidence in the result.

**Table S3.** Reported adverse events among the included studies, n (%).

| **Study ID** | **Li et al. 2022 (4)** | | **Lu et al. 2021 (2)** | | **Wang et al. 2021 (3)** | | | **Zhou et al. 2022 (1)** | |
| --- | --- | --- | --- | --- | --- | --- | --- | --- | --- |
| **Groups** | **TS + CT** | **CT** | **TS + CT** | **CT** | **TS + CT (PTX + CBP)** | **TS + CT (nab PTX + CBP)** | **CT (PTX + CBP)** | **TS** | **CT** |
| **Constipation** | N/A | N/A | Grades 1–2: 53 (23.9)  Grades ≥ 3: 0 (0.0) | Grades 1–2: 24 (21.8)  Grades ≥ 3: 0 (0.0) | All grades: 36 (30.0)  Grade ≥3: 0 (0.0) | All grades: 33 (28.0)  Grade ≥3: 0 (0.0) | All grades: 27 (23.1)  Grade ≥3: 0 | All grades: 73 (13.7)  Grade ≥3: 0 (0.0) | All grades: 43 (16.7)  Grade ≥3: 0 (0.0) |
| **Pneumonia** | N/A | N/A | N/A | N/A | N/A | N/A | N/A | All grades: 71 (13.3)  Grade ≥3: 40 (7.5) | All grades: 36 (14.0)  Grade ≥3: 24 (9.3) |
| **Hypoproteinemia** | N/A | N/A | Grades 1–2: 51 (23.0)  Grades ≥ 3: 0 (0.0) | Grades 1–2: 15 (13.6)  Grades ≥ 3: 0 (0.0) | N/A | N/A | N/A | N/A | N/A |
| **Hypoalbuminemia** | N/A | N/A | N/A | N/A | All grades: 27 (22.5)  Grade ≥3: 1 (0.8) | All grades: 21 (17.8)  Grade ≥3: 0 (0.0) | All grades: 19 (16.2)  Grade ≥3: 0 (0.0) | All grades: 76 (14.2)  Grade ≥3: 1 (0.2) | All grades: 40 (15.5)  Grade ≥3: 1 (0.4) |
| **Nausea** | N/A | N/A | Grades 1–2: 96 (43.2)  Grades ≥ 3: 1 (0.5) | Grades 1–2: 44 (40.0)  Grades ≥ 3: 1 (0.9) | All grades: 36 (30.0)  Grade ≥3: 0 (0.0) | All grades: 51 (43.2)  Grade ≥3: 0 (0.0) | All grades: 35 (29.9)  Grade ≥3: 1 (0.9) | All grades: 61 (11.4)  Grade ≥3: 0 (0.0) | All grades: 43 (16.7)  Grade ≥3: 1 (0.4) |
| **Fatigue** | N/A | N/A | Grades 1–2: 82 (36.9)  Grades ≥ 3: 3 (1.4) | Grades 1–2: 38 (34.5)  Grades ≥ 3: 2 (1.8) | N/A | N/A | N/A | N/A | N/A |
| **Decreased weight** | N/A | N/A | N/A | N/A | N/A | N/A | N/A | All grades: 86 (16.1)  Grade ≥3: 4 (0.7) | All grades: 30 (11.6)  Grade ≥3: 0 (0.0) |
| **Vomiting** | N/A | N/A | Grades 1–2: 59 (26.6)  Grades ≥ 3: 1 (0.5) | Grades 1–2: 24 (21.8)  Grades ≥ 3: 1 (0.9) | All grades: 28 (23.3)  Grade ≥3: 1 (0.8) | All grades: 27 (22.9)  Grade ≥3: 0 (0.0) | All grades: 20 (17.1)  Grade ≥3: 2 (1.7) | N/A | N/A |
| **Musculoskeletal pain** | N/A | N/A | Grades 1–2: 55 (24.8)  Grades ≥ 3: 0 (0.0) | Grades 1–2: 19 (17.3)  Grades ≥ 3: 2 (1.8) | N/A | N/A | N/A | N/A | N/A |
| **Hyponatremia** | N/A | N/A | N/A | N/A | N/A | N/A | N/A | All grades: 52 (9.7)  Grade ≥3: 10 (1.9) | All grades: 29 (11.2)  Grade ≥3: 11 (4.3) |
| **Hyperglycemia** | N/A | N/A | N/A | N/A | N/A | N/A | N/A | All grades: 55 (10.3)  Grade ≥3: 8 (1.5) | All grades: 29 (11.2)  Grade ≥3: 3 (1.2) |
| **Hemoptysis** | N/A | N/A | N/A | N/A | N/A | N/A | N/A | All grades: 59 (11.0)  Grade ≥3: 6 (1.1) | All grades: 22 (8.5)  Grade ≥3: 3 (1.2) |
| **Weakness** | 15 (45.45) | 13 (39.39) | N/A | N/A | N/A | N/A | N/A | N/A | N/A |
| **Insomnia** | N/A | N/A | N/A | N/A | N/A | N/A | N/A | All grades: 33 (6.2)  Grade ≥3: 1 (0.2) | All grades: 26 (10.1)  Grade ≥3: 0 (0.0) |
| **Mouth ulcer** | 14 (42.42) | 12 (36.36) | N/A | N/A | N/A | N/A | N/A | N/A | N/A |
| **Alopecia** | 15 (45.45) | 18 (54.55) | N/A | N/A | All grades: 77 (64.2)  Grade ≥3: 0 (0.0) | All grades: 82 (69.5)  Grade ≥3: 0 (0.0) | All grades: 72 (61.5)  Grade ≥3: 0 | All grades: 7 (1.3)  Grade ≥3: 0 (0.0) | All grades: 126 (48.8)  Grade ≥3: 2 (0.8) |
| **Cough** | N/A | N/A | N/A | N/A | N/A | N/A | N/A | All grades: 114 (21.3)  Grade ≥3: 5 (0.9) | All grades: 40 (15.5)  Grade ≥3: 1 (0.4) |
| **Hypothyroidism** | N/A | N/A | N/A | N/A | N/A | N/A | N/A | All grades: 62 (11.6)  Grade ≥3: 0 (0.0) | All grades: 2 (0.8)  Grade ≥3: 0 (0.0) |
| **Pyrexia** | N/A | N/A | N/A | N/A | All grades: 24 (20.0)  Grade ≥3: 0 (0.0) | All grades: 24 (20.3)  Grade ≥3: 1 (0.8) | All grades: 18 (15.4)  Grade ≥3: 0 | All grades: 61 (11.4)  Grade ≥3: 2 (0.4) | All grades: 26 (10.1)  Grade ≥3: 0 (0.0) |
| **Dyspnea** | N/A | N/A | N/A | N/A | N/A | N/A | N/A | All grades: 67 (12.5)  Grade ≥3: 11 (2.1) | All grades: 36 (14.0)  Grade ≥3: 7 (2.7) |
| **Diarrhea** | N/A | N/A | N/A | N/A | N/A | N/A | N/A | All grades: 40 (7.5)  Grade ≥3: 4 (0.7) | All grades: 35 (13.6)  Grade ≥3: 5 (1.9) |
| **Asthenia** | N/A | N/A | N/A | N/A | All grades: 29 (24.2)  Grade ≥3: 0 (0.0) | All grades: 21 (17.8)  Grade ≥3: 0 (0.0) | All grades: 24 (20.5)  Grade ≥3: 0 (0.0) | All grades: 75 (14.0)  Grade ≥3: 7 (1.3) | All grades: 58 (22.5)  Grade ≥3: 14 (5.4) |
| **Increased blood bilirubin levels** | N/A | N/A | N/A | N/A | All grades: 27 (22.5)  Grade ≥3: 0 (0.0) | All grades: 15 (12.7)  Grade ≥3: 0 (0.0) | All grades: 15 (12.8)  Grade ≥3: 0 (0.0) | N/A | N/A |
| **Hypoesthesia** | N/A | N/A | N/A | N/A | All grades: 27 (22.5)  Grade ≥3: 0 (0.0) | All grades: 12 (10.2)  Grade ≥3: 0 (0.0) | All grades: 19 (16.2)  Grade ≥3: 0 (0.0) | N/A | N/A |
| **Arthralgia** | N/A | N/A | N/A | N/A | All grades: 25 (20.8)  Grade ≥3: 0 (0.0) | All grades: 21 (17.8)  Grade ≥3: 0 (0.0) | All grades: 19 (16.2)  Grade ≥3: 0 (0.0) | All grades: 68 (12.7)  Grade ≥3: 1 (0.2) | All grades: 24 (9.3)  Grade ≥3: 1 (0.4) |
| **Pain in extremity** | N/A | N/A | N/A | N/A | All grades: 40 (33.3)  Grade ≥3: 3 (2.5) | All grades: 17 (14.4)  Grade ≥3: 0 (0.0) | All grades: 27 (23.1)  Grade ≥3: 0 (0.0) | N/A | N/A |
| **Febrile neutropenia** | N/A | N/A | N/A | N/A | N/A | N/A | N/A | All grades: 0 (0.0)  Grade ≥3: 0 (0.0) | All grades: 33 (12.8)  Grade ≥3: 33 (12.8) |
| **Decreased neutrophil levels** | N/A | N/A | N/A | N/A | All grades: 76 (63.3)  Grade ≥3: 62 (51.7) | All grades: 72 (61.0)  Grade ≥3: 54 (45.8) | All grades: 68 (58.1)  Grade ≥3: 53 (45.3) | All grades: 16 (3.0)  Grade ≥3: 3 (0.6) | All grades: 95 (36.8)  Grade ≥3: 71 (27.5) |
| **Decreased WBC count** | N/A | N/A | N/A | N/A | All grades: 64 (53.3)  Grade ≥3: 27 (22.5) | All grades: 68 (57.6)  Grade ≥3: 32 (27.1) | All grades: 62 (53.0)  Grade ≥3: 28 (23.9) | All grades: 20 (3.7)  Grade ≥3: 1 (0.2) | All grades: 74 (28.7)  Grade ≥3: 47 (18.2) |
| **Decreased PLT count** | N/A | N/A | N/A | N/A | All grades: 41 (34.2)  Grade ≥3: 5 (4.2) | All grades: 52 (44.1)  Grade ≥3: 16 (13.6) | All grades: 28 (23.9)  Grade ≥3: 2 (1.7) | N/A | N/A |
| **Rash** | 14 (42.42) | 13 (39.39) | Grades 1–2: 46 (20.7)  Grades ≥ 3: 1 (0.5) | Grades 1–2: 13 (11.8)  Grades ≥ 3: 0 (0.0) | All grades: 25 (20.8)  Grade ≥3: 4 (3.3) | All grades: 26 (22.0)  Grade ≥3: 2 (1.7) | All grades: 4 (3.4)  Grade ≥3: 0 (0.0) | N/A | N/A |
| **Decreased appetite** | N/A | N/A | Grades 1–2: 72 (32.4)  Grades ≥ 3: 3 (1.4) | Grades 1–2: 32 (29.1)  Grades ≥ 3: 1 (0.9) | All grades: 52 (43.3)  Grade ≥3: 1 (0.8) | All grades: 52 (44.1)  Grade ≥3: 1 (0.8) | All grades: 36 (30.8)  Grade ≥3: 1 (0.9) | All grades: 88 (16.5)  Grade ≥3: 5 (0.9) | All grades: 62 (24.0)  Grade ≥3: 3 (1.2) |
| **Leukopenia** | N/A | N/A | Grades 1–2: 135 (60.8)  Grades ≥ 3: 48 (21.6) | Grades 1–2: 65 (59.1)  Grades ≥ 3: 16 (14.5) | All grades: 57 (47.5)  Grade ≥3: 19 (15.8) | All grades: 66 (55.9)  Grade ≥3: 30 (25.4) | All grades: 56 (47.9)  Grade ≥3: 21 (17.9) | All grades: 17 (3.2)  Grade ≥3: 1 (0.2) | All grades: 73 (28.3)  Grade ≥3: 41 (15.9) |
| **Increased ALT** | N/A | N/A | Grades 1–2: 100 (45.0)  Grades ≥ 3: 8 (3.6) | Grades 1–2: 47 (42.7)  Grades ≥ 3: 3 (2.7) | All grades: 50 (41.7)  Grade ≥3: 2 (1.7) | All grades: 41 (34.7)  Grade ≥3: 2 (1.7) | All grades: 27 (23.1)  Grade ≥3: 0 (0.0) | All grades: 110 (20.6)  Grade ≥3: 5 (0.9) | All grades: 39 (15.1)  Grade ≥3: 0 (0.0) |
| **Thrombocytopenia** | 6 (18.18) | 7 (21.21) | Grades 1–2: 112 (50.5)  Grades ≥ 3: 43 (19.4) | Grades 1–2: 55 (50.0)  Grades ≥ 3: 15 (13.6) | All grades: 33 (27.5)  Grade ≥3: 7 (5.8) | All grades: 47 (39.8)  Grade ≥3: 15 (12.7) | All grades: 32 (27.4)  Grade ≥3: 7 (6.0) | N/A | N/A |
| **Increased AST** | N/A | N/A | Grades 1–2: 91 (41.0)  Grades ≥ 3: 5 (2.3) | Grades 1–2: 51 (46.4)  Grades ≥ 3: 0 (0.0) | All grades: 43 (35.8)  Grade ≥3: 0 (0.0) | All grades: 40 (33.9)  Grade ≥3: 1 (0.8) | All grades: 14 (12.0)  Grade ≥3: 0 (0.0) | All grades: 104 (19.5)  Grade ≥3: 5 (0.9) | All grades: 32 (12.4)  Grade ≥3: 1 (0.4) |
| **Anemia** | 5 (15.15) | 6 (18.18) | Grades 1–2: 158 (71.2)  Grades ≥ 3: 33 (14.9) | Grades 1–2: 79 (71.8)  Grades ≥ 3: 13 (11.8) | All grades: 106 (88.3)  Grade ≥3: 9 (7.5) | All grades: 110 (93.2)  Grade ≥3: 27 (22.9) | All grades: 94 (80.3)  Grade ≥3: 14 (12.0) | All grades: 156 (29.2)  Grade ≥3: 18 (3.4) | All grades: 115 (44.6)  Grade ≥3: 18 (7.0) |
| **Neutropenia** | 12 (36.36) | 11 (33.33) | N/A | N/A | All grades: 51 (42.5)  Grade ≥3: 40 (33.3) | All grades: 50 (42.4)  Grade ≥3: 32 (27.1) | All grades: 55 (47.0)  Grade ≥3: 47 (40.2) | All grades: 10 (1.9)  Grade ≥3: 3 (0.6) | All grades: 81 (31.4)  Grade ≥3: 72 (27.9) |

Abbreviations: TS: tislelizumab; CT: chemotherapy; PTX: paclitaxel; CBP: carboplatin; WBC: white blood cell; PLT: platelet; ALT: alanine transaminase; AST: aspartate transaminase; N/A: not available.

**References**

1. Zhou C, Huang D, Fan Y, Yu X, Liu Y, Shu Y, et al. Tislelizumab Versus Docetaxel in Patients With Previously Treated Advanced NSCLC (RATIONALE-303): A Phase 3, Open-Label, Randomized Controlled Trial. Journal of Thoracic Oncology. 2022.

2. Lu S, Wang J, Yu Y, Yu XM, Hu YP, Ai XH, et al. Tislelizumab Plus Chemotherapy as First-Line Treatment for Locally Advanced or Metastatic Nonsquamous NSCLC (RATIONALE 304): A Randomized Phase 3 Trial. Journal of Thoracic Oncology. 2021;16(9):1512-22.

3. Wang J, Lu S, Yu XM, Hu YP, Sun YP, Wang ZJ, et al. Tislelizumab Plus Chemotherapy vs Chemotherapy Alone as First-line Treatment for Advanced Squamous Non-Small-Cell Lung Cancer A Phase 3 Randomized Clinical Trial. Jama Oncology. 2021;7(5):709-17.

4. Li L, Zhu Y, Lu W, Lin W, Guo N, Chen M. Short-Term and Long-Term Efficacy and Safety of Pemetrexed and Tislelizumab in Advanced Epidermal Growth Factor Receptor Tumor Protein 53 Co-Variant Lung Adenocarcinoma. Indian Journal of Pharmaceutical Sciences. 2022:92-6.
